# Functional Analysis of the Halastavi árva Virus (HalV) Internal Ribosome Entry Site

**DOI:** 10.3390/v18050492

**Published:** 2026-04-23

**Authors:** Subash Chapagain, Lauren F. Woodburn, Natalie C. J. Strynadka, Eric Jan

**Affiliations:** Department of Biochemistry and Molecular Biology, Life Sciences Institute, University of British Columbia, Vancouver, BC V6T 1Z4, Canada; chsubash@student.ubc.ca (S.C.); lauren.woodburn@ubc.ca (L.F.W.); natalie.strynadka@ubc.ca (N.C.J.S.)

**Keywords:** IRES, ribosome, RNA, virus, tRNA

## Abstract

Viral internal ribosome entry sites (IRESs) are specialized RNA structures that facilitate cap-independent translation as a strategy to usurp the host translational machinery. The Type 6 IRESs are the most streamlined mechanism to date, as they adopt a three pseudoknot RNA structure to initiate factorless translation initiation by directly recruiting the ribosome and drive translation. The Halastavi árva virus (HalV) IRES represents the most minimalistic subclass identified to date, whereby the IRES lacks specific pseudoknot domains that bind to the 40S subunit but instead recruits pre-assembled 80S ribosomes via a mechanism that is not fully understood. Here, we examined cellular conditions that can support HalV IRES translation. We demonstrated that the HalV IRES is translationally active in insect Sf21 lysates and *Drosophila* S2 cells, but inactive in mammalian RRL and wheat germ extract. Cells treated with heat shock or serum starvation suppressed HalV IRES activity, whereas virus infection robustly enhanced HalV IRES-mediated translation. Finally, the HalV IRES can support viral translation and replication using a heterologous viral replicon. These findings highlight the context-specific cellular conditions that allow ribosome assembly and translation by a factorless minimalist IRES.

## 1. Introduction

RNA viruses have evolved diverse mechanisms to commandeer the host translational machinery to drive viral protein synthesis for productive infection [1]. One such mechanism is the internal ribosome entry site (IRES) mechanism that is utilized by some positive-sense RNA viruses, including in the viral families *Picornaviridae*, *Calciviridae* and *Dicistroviridae* [2]. Unlike cap-dependent translations that are used by most eukaryotic mRNAs, IRESs adopt RNA structures that direct 5′ cap-independent translation initiation [3,4]. IRESs are functionally defined and identified by whether an RNA element can support internal ribosome entry and translation generally using a standard bicistronic RNA or circular RNA reporter. IRESs typically use a subset of translation initiation factors and may use specific host factors called IRES trans-acting factors (ITAFs) to promote translation [5,6]. Viral IRESs are proposed to facilitate preferential translation during infection, especially when cap-dependent translation is inhibited [7,8].

Based on factor requirements and conserved RNA structure and domains, viral IRESs are categorized into specific types, of which there are currently 6 types; however, it is likely that more classes will be identified with the expansion of viromes identified [9,10,11]. Type 1 and 2 IRESs, typified by poliovirus and encephalomyocarditis (EMCV), respectively, require most of the translation initiation factors and their activity is enhanced by host IRES trans-acting factors like polypyrimidine binding protein (PTB1) [12,13,14,15]. Type 1 IRESs involve 40S scanning to the AUG start codon whereas Type 2 IRESs directly recruit 40S near the AUG start codon. Type 3 IRESs, such as the hepatitis A virus (HAV) IRES, require the cap-binding proteins eIF4E and eIF4G [16,17]. Type 4 IRESs, exemplified by hepatitis C virus (HCV) and classic swine fever virus (CSFV) IRESs, can recruit the 40S ribosomal subunit directly and use only eIF3 and eIF2 for translation initiation [18,19,20]. Type 5 IRESs, found in the genomes of Aichiviruses, have adopted sub-domains of and have similar factor requirements to Type 1 and 2 IRESs, and require the host protein DHX29 to initiate translation [21,22].

The Type 6 IRESs in the genomes of the *Dicistroviridae* intergenic region (IGR) are the most unique due to their RNA structural autonomy and ability to directly bind to the ribosome, initiating translation in a factorless and initiator tRNA-independent manner from a non-AUG codon [23,24,25,26,27,28]. The intergenic region IRES (IGR IRES) in Cricket paralysis virus (CrPV), honeybee-infecting Israeli acute paralysis virus (IAPV) and Taura syndrome virus (TSV) are examples of this IRES class. Type 6 IRESs are ~160–180 nucleotides in length, and in general adopt four stem-loops with three overlapping pseudoknots [25,29,30,31]. Biochemical and cryo-EM structures have uncovered the mechanistic details of how these IRESs use two independent domains to recruit ribosomes and drive translation: PKII and PKIII bind to the 40S and 60S subunits while PKI mimics a tRNA anticodon–codon interaction [32,33]. Spanning all three tRNA binding sites, the IGR IRES initially binds to the intersubunit space of the ribosome, whereby the PKI tRNA anticodon mimicry domain occupies the ribosomal A site and is then translocated to the P site by eEF2 [28,31]. IGR IRES-driven translation persists under cellular stresses that normally suppress cap-dependent translation [34,35]. The efficiency and functional relevance of IGR IRESs have been confirmed in vivo in a viral context, with mutational studies highlighting the necessity of specific structural elements for both ribosome binding and viral propagation [34,36,37,38,39].

Type 6 IRESs are further classified into distinct subtypes based on their specific RNA structures and mechanisms of action [40]. Type 6a and 6b IRESs are typified by the CrPV and IAPV IRESs respectively where the main difference is that the IAPV IRES harbors an extra stem-loop (SLIII) within the PKI domain, apart from specifically conserved L1.1 sequences for each [41]. Despite these differences, both Type 6a and 6b IRESs use similar mechanisms for ribosomal interaction and translation initiation. PKI domains of these IGR IRESs can be swapped while retaining translational activity [42,43]. Type 6c-6f IRESs are distinct in that they can recruit pre-formed 80S ribosomes despite each having distinct substructures—each subtype has distinct PKII and PKIII domains, but all contain a PKI codon–anticodon mimicry domain [44,45,46]. For instance, Type 6d-6f IRESs contain a typical PKII base pair, but the SLV domain is shorter than in Type 6a and 6b and the PKIII base pair is contained within the apical stem of SLV.

Type 6c IRES, identified in the genome of Halastavi árva virus (HalV) [47], is the most simplistic IGR IRES reported till date [46]. Unlike Type 6a and 6b IRESs, the HalV IGR IRES lacks the SLV, SLIV, and PKIII domains, yet can bind to 80S ribosomes with its PKI domain positioned in the ribosomal P site. As a consequence, this IRES bypasses the requirement for eEF2-mediated translocation of PKI from the A site to the P site that is characteristic of Type 6a and 6b IRESs [46]. Notably, HalV IGR IRES-mediated ribosome binding and translation are inhibited in rabbit reticulocyte lysates by the SERBP1/eEF2 complex, which associates with non-programmed 80S ribosomes [46]. These observations point to a distinct ribosome assembly pathway and raise fundamental questions regarding how the HalV IGR IRES recruits ribosomes to direct viral protein synthesis, as well as whether similar assembly pathways operate in other cellular contexts. In this regard, recent metagenomic and functional studies have uncovered diverse viral RNA strategies for translation, suggesting that factorless translation initiation mechanisms are more prevalent than previously appreciated and may be selectively enabled by specific host cellular environments during infection [10,40,48]. In this study, we define the cellular contexts in which the HalV IGR IRES can productively engage the host translation machinery. Using a bicistronic reporter assay, we show that HalV IGR IRES-mediated translation is selectively enhanced during CrPV infection and remains partially refractory to ER stress-induced translational repression, but is strongly inhibited under heat shock or serum starvation conditions. Moreover, we demonstrate that the HalV IGR IRES supports translation in the context of a dicistrovirus replicon, providing a functional readout of IRES-driven protein synthesis coupled to viral RNA replication. Together, these results define cellular contexts that permit 80S ribosome assembly on the HalV IGR IRES, offering insights into this highly streamlined, factor-independent mode of translation.

## 2. Materials and Methods

### 2.1. Cell Culture

*Drosophila* Schneider 2 cell line (S2) cells (Gibco, Carlsbad, CA, USA; Cat # 51-4003) were maintained in Shields and Sang M3 insect medium (Sigma-Aldrich, St. Louis, MO, USA) supplemented with 10% fetal bovine serum (FBS) at 25 °C unless indicated otherwise.

### 2.2. Plasmids

HaIV IGR IRES fragments (nucleotides 6277 to nucleotides 6408, Accession No. MN231041) were synthesized (Twist Biosciences, San Francisco, CA, USA) and cloned into bicistronic and luciferase reporter plasmids using Gibson assembly. Deletion mutations and specific nucleotide mutations were generated by site-directed mutagenesis and sequence-verified (Genewiz, Seattle, WA, USA and Plasmidsaurus, Seattle, WA, USA).

CrPV and HaIV IGR-IRES-Nanoluc replicons were generated by modifying a previously published CrPV infection clone, CrPV-3 [34]. Briefly, a fragment encoding CrPV IGR-Nanoluciferase or HalV IGR-Nanoluciferase (Twist Biosciences) was inserted into the CrPV-3 infectious clone where the ORF2 was replaced by either CrPV IGR-IRES-Nanoluciferase or HalV IGR-IRES-Nanoluciferase. In both cases, the AUG start codon of the Nanoluciferase ORF was deleted. RdRp catalytic-inactive mutant versions of both the CrPV and HalV replicon (DD1620-1 to NN) were generated by site-directed mutagenesis and sequence-verified (Plasmidsaurus).

### 2.3. In Vitro Transcription

Bicistronic and replicon plasmids were linearized by BamHI and Eco53KI (NEB, Ipswich, MA, USA) restriction enzymes, respectively. RNA was transcribed using T7 RNA polymerase and subsequently purified with a Monarch RNA extraction Kit (NEB). 5′capping and polyadenylation were performed post-transcriptionally (CellScript, Madison, WI, USA). The RNA integrity and purity were confirmed by denaturing agarose gel analysis, and the concentration was measured with a spectrophotometer (Nanodrop, Thermo Fisher Scientific, Wilmington, DE, USA).

### 2.4. Transfection

Drosophila Schneider S2 cells (2.5 × 10^6^ cells) were transfected with bicistronic or replicon RNA (2 μg) using Lipofectamine 2000 reagent (Thermo Fisher Scientific, Waltham, MA, USA). S2 cells were transfected with bicistronic RNA for 1 h, followed by infection with CrPV (MOI = 10) for 4.5 h to monitor reporter RNA translation. For replicon transfection experiments, S2 cells were transfected and then harvested at the indicated times and lysed in 1× passive lysis buffer (Promega, Madison, WI, USA). Lysates were cleared and the protein concentration was measured by Bradford assay (Bio-Rad, Hercules, CA, USA).

### 2.5. In Vitro Translation Reactions

In vitro-transcribed bicistronic reporter RNA (1 µg) was incubated in Sf-21 cell extract (Promega), wheat germ extract (WGE), or rabbit reticulocyte lysate (RRL) (Promega) for 1.5 h (WGE) or 1 h (RRL). Luciferase activities were measured (Promega) using a Tecan Spark-10M Multimode plate reader.

### 2.6. Purification of the 40S and 60S Subunits

HeLa cell pellets (Cell Culture Company, Shoreview, MN, USA) were used to purify ribosomes as previously described [49]. Briefly, cells were lysed in a mild lysis buffer consisting of 15 mM Tris-HCL (pH 7.5), 300 mM NaCl, 2 mM Mg(OAc)2, 1 mM DTT, 0.5% (*v*/*v*) NP-40, and 0.1 U/μL RNAse inhibitor. Debris was removed by centrifuging at 12,000× *g* and the supernatant was layered with a 30% (*w*/*w*) cushion of sucrose in 150 mM KCl and centrifuged for 16 h at 115,800× *g* to pellet crude ribosomes. Pelleted ribosomes were gently resuspended in buffer B (20 mM Tris-HCL (pH 7.5), 6 mM magnesium acetate, 150 mM KCL, 6.8% (*w*/*v*) sucrose, 1 mM DTT) at 4 °C. Next, the ribosomes were treated with puromycin (final concentration 2.3 mM) to release mRNA and KCl (final concentration 500 mM) was added to wash and dissociate 80S ribosomes into 40S and 60S. The dissociated ribosomes were then separated on 20–40% (*w*/*w*) linear sucrose gradients prepared in BioComp Gradient Station (Biocomp Instruments Ltd, Tatamagouche, NS, Canada). Absorbance at 260 nm was measured to detect the 40S and 60S peaks and corresponding fractions were pooled and concentrated using Amicon Ultra spin concentrators (Millipore Sigma, Burlington, MA, USA) in buffer C (20 mM Tris-HCl (pH 7.5), 0.2 mM ED buy TA, 10 mM KCL, 1 mM MgCl_2_, 6.8% sucrose). The conversions 1 A260 nm  =  50 nM for 40S subunits and 1 A260 nm  =  25 nM for 60S subunits were used to determine the concentration of 40S and 60S subunits by spectrophotometry.

### 2.7. Ribosome Filter Binding Assays

A filter binding assay was used to analyze ribosome:IRES complexes as described [42]. Briefly, in vitro-transcribed RNAs were dephosphorylated with Antarctic phosphatase (NEB) then labeled with T4 polynucleotide kinase (NEB) and [γ^32^P]-ATP. [γ^32^P]-RNA (final concentration: 0.5 nM) was heated at 65 °C for 3 min prior to the addition of 1× buffer E (final concentration: 20 mM Tris pH 7.5, 100 mM KCl, 2.5 mM MgOAc, 0.25 mM Spermidine and 2 mM DTT) and gently cooled to room temperature for 20 min, allowing for proper folding. The RNA was then incubated with 40S ribosomal subunits from 0.1 nM to 100 nM for 15 min. For 80S assembly/binding, 40S and 60S ribosomal subunits were added from 0.15 nM to 150 nM for 15 min, with 50 ng/mL in vitro-transcribed non-competitor RNA prepared from pcDNA3 (linearized with EcoRV). Bio-Dot apparatus (Bio-Rad, Hercules, CA, USA) was used to vacuum-filter the reactions with nitrocellulose (top) and nylon (bottom) membranes. The membranes were washed thrice with 1X buffer E before drying and analyzed by a phosphorimager (ImageQuant™ 800, Amersham Typhoon, Marlborough, MA, USA). The fraction bound and the dissociation constant (K_D_) were calculated as previously described [50].

### 2.8. Metabolic Radiolabeling

S2 cells with mock or CrPV (MOI 10) were infected for 6 h followed by incubation with 50 μCi of Easy-Tag Express [^35^S]protein labeling mix (Perkin-Elmer, Waltham, MA, USA)/mL for 30 min. Cells were washed in cold 1× PBS and lysed in lysis buffer (20 mM HEPES, 150 mM NaCl, 1% Triton X-100, 10% glycerol, 1 mM EDTA, 10 mM tetrapyrophosphate, 100 mM NaF, 17.5 mM β-glycerophosphate, and a protease inhibitor cocktail (Roche, South San Francisco, CA, USA). Protein concentration was determined by Bradford assay (Bio-Rad), and equal amounts (20 μg) of lysates were separated by 12% SDS-PAGE. Gels were dried and imaged using a Typhoon imager (ImageQuant™ 800) for analysis.

### 2.9. RT-qPCR of Replicon RNA

Total RNA was extracted from transfected Drosophila S2 cells at 3 and 18 h post-transfection by Trizol (Thermo Fisher Scientific). Extracted RNA was DNase-treated for 30 min at 37 °C. First-strand cDNA synthesis was performed using a Lunascript RT Supermix Kit (NEB) following the manufacturer’s protocol. Quantitative real-time PCR (qPCR) was subsequently performed using Luna Universal qPCR Master Mix (NEB) on an QuantStudio real-time PCR system (Applied Biosystems, South San Francisco, CA, USA). Target amplification of the NLuc reporter sequence within the replicons was performed using the primers: forward, 5′-GGCTACAACCTGGACCAAGT-3′ and reverse, 5′-ACGGGATGATGACATGGATG-3′. For internal normalization, the house-keeping gene eS6 was amplified as a reference control using the primers: forward, 5′-CGATATCCTCGGTGACGAGT-3′ and reverse, 5′-CCCTTCTTCAAGACGACCAG-3′. No-RT and no-template controls were used to confirm the absence of non-specific amplification. Relative RNA levels were calculated using the 2^−ΔΔCt^ method, where the target C_t_ values were first normalized to the C_t_ of the eS6 reference gene (ΔC_t_). These values were then normalized to wild-type RNA levels at 3 h for each, defined as 1.0.

### 2.10. Statistical Analysis

We used GraphPad PRISM version 10 software to analyze data. All data are presented as mean ± standard deviation (s.d.) from at least three independent biological experiments. For luciferase reporter assays comparing IRES variants, data were analyzed by one-way analysis of variance (ANOVA) followed by Tukey’s multiple comparisons test. For stress experiments and CrPV infection studies, mock and treated conditions were compared for each IRES variant using paired two-tailed Student’s *t*-tests. For RT-qPCR analysis, fold change values calculated by the ΔΔC_t_ method were compared between wild-type (WT) and replicase-deficient (mRdRP) replicons using paired two-tailed Student’s *t*-tests at each time point. Differences were considered statistically significant at *p* < 0.05.

## 3. Results

### 3.1. HaIV IGR IRES Is Translationally Active in Insect Lysates and Drosophila S2 Cells

**Figure 1:**
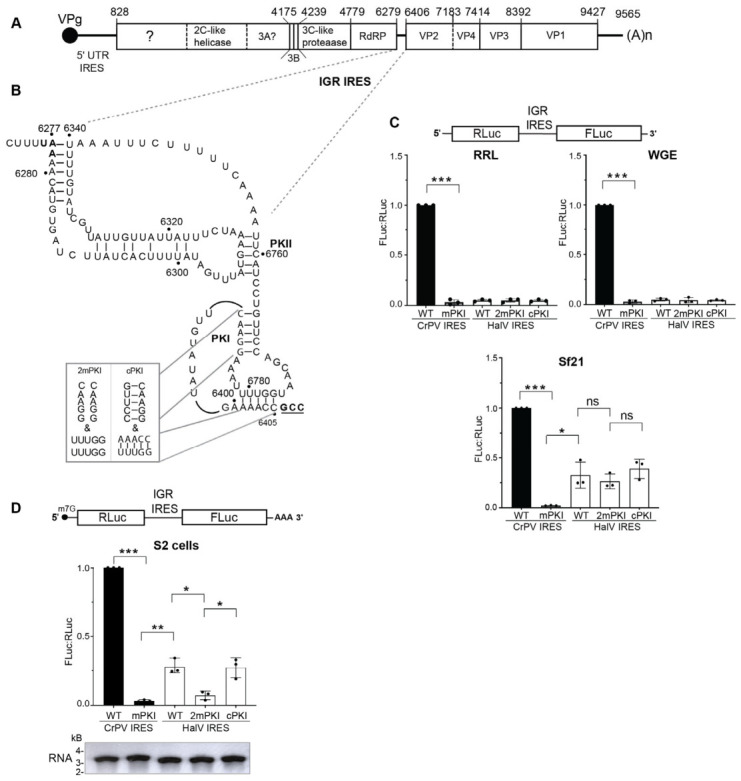
Translational activity of HalV IGR IRES in vitro. (**A**) Schematic of the HalV genome. (**B**) Secondary structure of the HalV IRES. Stop codon of the upstream ORF1 is in bold and start codon of downstream ORF2 is bold, underlined. Mutations that disrupt both the stem and the pseudoknot base pairs within PKI domain (2mPKI) and compensatory mutations (cPKI) to restore the base pairing are shown. (**C**) Bicistronic reporter RNA containing either the wild-type or mutant PKI (mPKI, cPKI) CrPV or HalV IGR IRESs were incubated in rabbit reticulocyte lysates (RRLs) (1 h, 37 °C), wheat germ extract (WGE) extract (1 h, 30 °C) and Sf21 insect lysates (1.5 h, 30 °C). FLuc/RLuc luciferase activity ratios were normalized to those of bicistronic RNAs containing the wild-type CrPV IGR IRES. (**D**) Translational activity of capped bicistronic reporter RNAs in *Drosophila* S2 cells. Below shows the integrity of in vitro capped and polyadenylated RNA. S2 cells were transfected with 5′capped reporter RNAs (schematic on top) containing the indicated wild-type and mutant IGR IRES and luciferase activities measured at 6 h post-transfection. A one-way ANOVA with Tukey’s multiple comparisons test was used to determine the *p* values (* *p* < 0.05, ** *p* < 0.01, *** *p* < 0.001; ns, not significant). Shown are averages ± s.d. from at least three independent experiments.

A previous study by Hellen and colleagues showed that the HalV IGR IRES failed to support translation in vitro in rabbit reticulocyte lysates (RRLs), an effect attributed to the SERBP1/eEF2 complex binding to the 80S ribosome that prevents IRES recruitment [46]. We investigated whether the HalV IGR IRES (herein HalV IGR IRES or HalV IRES) can drive translation in other in vitro wheat germ and insect Sf-21 translation extracts. We used bicistronic reporter RNAs, which monitor scanning-dependent Renilla luciferase (RLuc) translation and IRES-mediated firefly luciferase (Fluc) translation, and then IRES activities were calculated from the relative luciferase FLuc:RLuc ratios (Figure 1C, Supplemental Data S1). As shown previously [51], the wild-type CrPV IGR IRES directed robust translation in all three extracts whereas a mutant variant containing mutations that disrupt all three PKs abolished translation (Figure 1C). Incubating bicistronic RNA containing the HalV IRES did not support IRES translation in the RRL, similar to that observed previously (Figure 1C) [46]. Also, HalV IRES was not active in wheat germ extract. By contrast, HalV IRES FLuc translation in Sf21 extracts was approximately 27% as compared to the wild-type CrPV IRES (Figure 1C). Combined disruption of base pairing of both the stem and the pseudoknot within the HalV PKI domain (2mPKI) slightly reduced IRES activity, although it was statistically insignificant, whereas compensatory mutations (cPKI) that restored PKI base pairing partially rescued IRES translation (Figure 1C). These results indicated that HalV IGR IRES can function in insect lysates in vitro.

Although the HaIV IGR IRES supported translation in vitro, the lack of dependence on the PKI integrity was unclear (Figure 1C). We next examined whether the HalV IGR IRES functions in S2 cells. We transfected in vitro-transcribed 5′m^7^G-capped bicistronic RNAs (Supplemental Figure S1A) into S2 cells and measured RLuc and FLuc activity at 6 h post-transfection. As expected, the wild-type but not the mutant mPKI CrPV IGR IRES supported FLuc translation in S2 cells (Figure 1D). The wild-type HalV IRES directed FLuc activity, ~20% of that of the CrPV IGR IRES whereas mutating both the stem and pseudoknot base pairing of the PKI mutant abolished HalV IRES FLuc translation (Figure 1D). By contrast, compensatory mutations that restored PKI base pairing rescued HalV IRES translation. Thus, in contrast to that observed in in vitro Sf21 extracts, the HaIV IGR IRES can support translation in S2 cells in a PKI-dependent manner.

### 3.2. HaIV IGR IRES Binds to Purified Human Ribosomes

**Figure 2:**
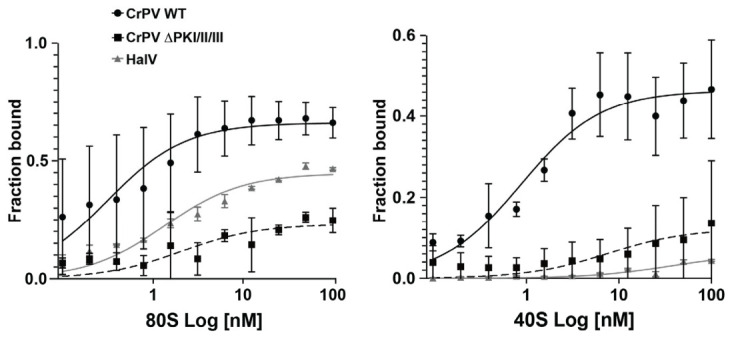
HaIV IRES:80S complexes. [^32^P]-HalV IGR IRES, [^32^P]-(CrPV) IGR IRES or mutant (∆PKI/II/III) CrPV IGR IRES were incubated with increasing amounts of purified salt-washed 40S/60S or 40S alone and then reactions were loaded onto a nitrocellulose/nylon filter binding assay. The filters were analyzed by phosphorimager analysis to quantify the fraction bound. Shown are the averages of at least three independent experiments ± s.d.

The HalV IRES lacks key stem-loops, SLIV and SLV, that are typically found in other dicistrovirus IGR IRESs and are necessary for 40S recruitment [46]. Thus, the HalV IGR IRES cannot recruit the rabbit 40S subunit but instead may recruit pre-assembled rabbit 80S ribosomes [46]. To examine this in more detail, we monitored 40S or 80S binding/assembly using an established filter binding assay and purified salt-washed human ribosomes [40]. Incubating an increasing concentration of purified 40S and 60S subunits with [^32^P]-end-labeled wild-type CrPV IRES resulted in CrPV IRES:ribosome complexes with an apparent K_D_ of 1.3 nM (Figure 2, circles), in line with previous reports [40]. In contrast, a mutant CrPV IRES with a disrupted base pairing in all three pseudoknots (ΔPKI/II/III) reduced ribosome binding (Figure 2, squares). Similarly, the HalV IRES bound to ribosomes with an apparent K_D_ of 2.3 nM (Figure 2, triangles). We next examined whether the HalV IRES binds to 40S subunits. Wild-type CrPV IGR IRES bound to 40S subunits with an apparent K_D_ of 2.3 nM; however, the triple pseudoknot mutant CrPV IGR IRES and the HalV IGR IRES showed much reduced or limited 40S binding (Figure 2). These findings support previous studies using rabbit ribosomes that the HalV IRES cannot bind to human 40S subunits but likely recruit pre-formed 80S ribosomes [46]. However, these experimental conditions do not preclude the possibility that 40S first binds to the HalV IRESs followed by 60S joining.

### 3.3. HalV IGR IRES Translation Under Cellular Stress Conditions

**Figure 3:**
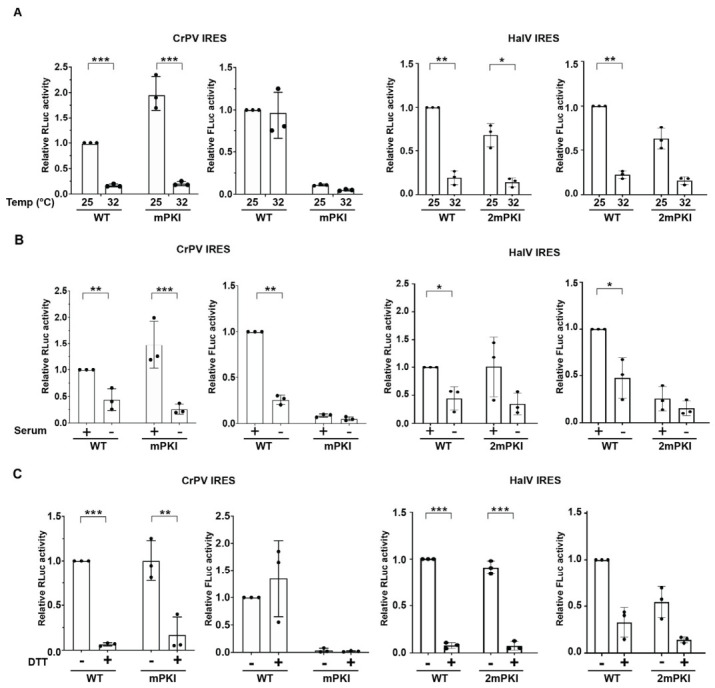
HalV IGR IRES translation under cellular stresses. S2 cells were transfected with bicistronic reporter RNAs (5′m^7^G capped and polyadenylated) containing the indicated wild-type or mutant IRES in untreated S2 cells or under (**A**) heat stress (32 °C, 3 h followed by 2 h recovery), (**B**) serum starvation (FBS-minus for 24 h prior to transfection), or (**C**) ER stress (4 mM, 4 h). Cells were harvested and luciferase activities were measured. Statistical differences between absolute luciferase units among stressed and unstressed conditions for each construct were assessed by paired two-tailed Student’s *t*-test. Only statistically significant differences are indicated by asterisks (* *p* < 0.05, ** *p* < 0.01, *** *p* < 0.001); all other comparisons were non-significant. All data are averages of ±s.d. from at least three independent experiments.

The HalV IGR IRES assembles pre-formed 80S ribosomes in vitro [46]; however, it is not clear whether this can occur in vivo and under specific cellular conditions that may accumulate empty pre-assembled 80S ribosomes. We systematically investigated whether HalV IGR IRES can support translation in S2 cells under specific cellular stresses, notably during heat shock (Figure 3A), serum starvation (Figure 3B), and ER stress (Figure 3C). We chose these cellular stresses as CrPV IGR IRES and other dicistrovirus IRES translations have been shown to be stimulated under these conditions [35,52]. Moreover, there is precedent that vacant 80S ribosomes may accumulate during nutrient deprivation—notably glucose starvation in yeast, and amino acid or serum starvation in mammalian cells [53,54]. We transfected IRES-containing 5′m^7^G-capped polyadenylated bicistronic RNAs into S2 cells in the presence and absence of cellular stress and measured RLuc and FLuc activities 6 h post-transfection. As expected, in all cellular stress conditions, cap-dependent RLuc translation was dramatically inhibited (by >90%), indicative of the global shutdown of protein synthesis. By contrast, wild-type CrPV IGR IRES FLuc translation was supported under heat shock and ER stress (DTT) conditions (Figure 3A,C), similar to that observed previously [52,55]. Interestingly, CrPV IGR IRES translation decreased under serum starvation conditions. In all cellular conditions, the mutant PKI CrPV IGR IRES, which disrupts only PKI base pairing, did not support IRES translation. The HalV IRES FLuc translation was inhibited or not supported in heat shock and serum-starved conditions, mirroring the decrease in cap-dependent RLuc translation (Figure 3A,B). However, under ER stress DTT-treated cells, HalV IGR IRES FLuc translation was partially supported (~25% compared to basal condition), which is in contrast to the dramatic decrease in cap-dependent translation (>90% decrease in DTT treated vs untreated cells). In sum, these results showed that HalV IGR IRES can support translation in *Drosophila* and human cells under ER stress.

### 3.4. HalV IGR IRES Activity Is Enhanced in CrPV-Infected S2 Cells

**Figure 4:**
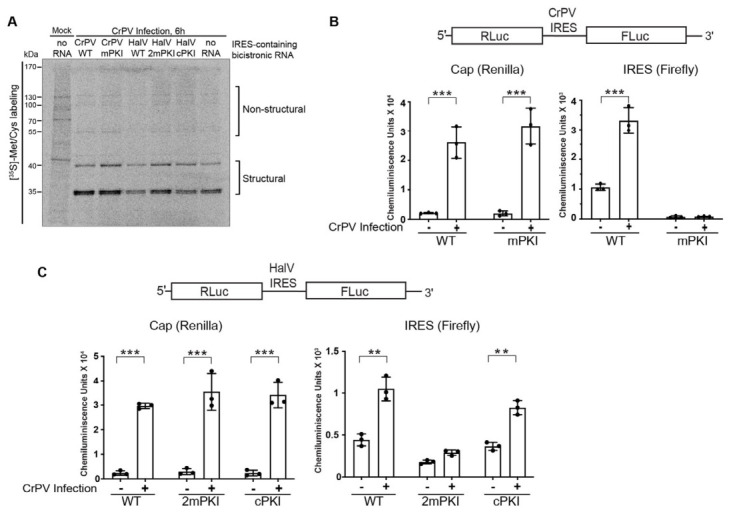
CrPV infection enhances HalV IGR IRES-mediated translation in S2 cells. (**A**) Phosphorimager analysis of protein lysates loaded on an SDS-12% PAGE gel. S2 cells were either mock- or CrPV-infected (MOI 10) for 1 h followed by transfection of indicated in vitro-transcribed 5′capped polyadenylated bicistronic RNAs. Cells were pulse-labeled with [^35^S] Met/Cys for 30 min at 6 h post-transfection (h.p.t.). In parallel, luciferase activities were measured for cells transfected with bicistronic reporter RNAs containing the wild-type (WT) or mutant (**B**) CrPV IRES or (**C**) HalV IRES. mPKI—single PKI base pair disruption (mPKI); 2mPKI—a double PKI base pair disruption mutant; cPKI—compensatory mutations that restored PKI base pairing. Absolute Renilla luciferase (cap-dependent translation, left) and firefly luciferase (IRES-mediated translation, right) are shown. Statistical differences between luciferase units in mock and infected conditions for each construct were assessed by paired two-tailed Student’s *t*-test. Only statistically significant differences are indicated by asterisks (** *p* < 0.01, *** *p* < 0.001). All data are averages of ± s.d. from at least three independent experiments.

During dicistrovirus infection, structural proteins are expressed in supramolar excess compared to non-structural proteins, indicating that the IGR IRES translation is stimulated during infection [55,56,57]. As an HalV virus infection cell model has not been established, we next tested whether HalV IGR IRES translation occurs in CrPV infection. We first monitored overall protein synthesis in mock- and CrPV-infected cells that were transfected with in vitro-transcribed IRES-containing capped bicistronic RNAs. Overall protein synthesis was monitored by [^35^S]-methioine pulse labeling for 30 min. In all cases, CrPV infection (6 h post-infection) resulted in the global shutdown of translation concomitant with CrPV structural and non-structural protein synthesis (Figure 4A) [55,56]. In parallel, we monitored luciferase activities. Cap-dependent Renilla translation was increased ~10-fold in mock- and CrPV-infected S2 cells (Figure 4B,C), which is consistent with observations reported previously [56]. Despite global translation shutoff in CrPV-infected cells as measured by 35^S^-methionine pulse labeling and polysome analysis [55], translation of transfected reporter RNAs was reproducibly observed in CrPV-infected S2 cells, which is attributed to a phenomenon that nascent mRNAs are privileged and stably translated under these cellular conditions [56,58]. Similarly, CrPV and HalV IGR IRES firefly luciferase translation were both stimulated under CrPV infection (Figure 4B,C). Disruption of pseudoknot base pairing abolished both CrPV and HalV IRES translation whereas compensatory mutations that restored PK base pairing rescued HalV IRES translation (Figure 4C), indicating that the integrity of the RNA structure is important for IRES translation in CrPV-infected cells.

### 3.5. HalV IGR IRES Supports Translation in a Heterologous Viral Replicon

**Figure 5:**
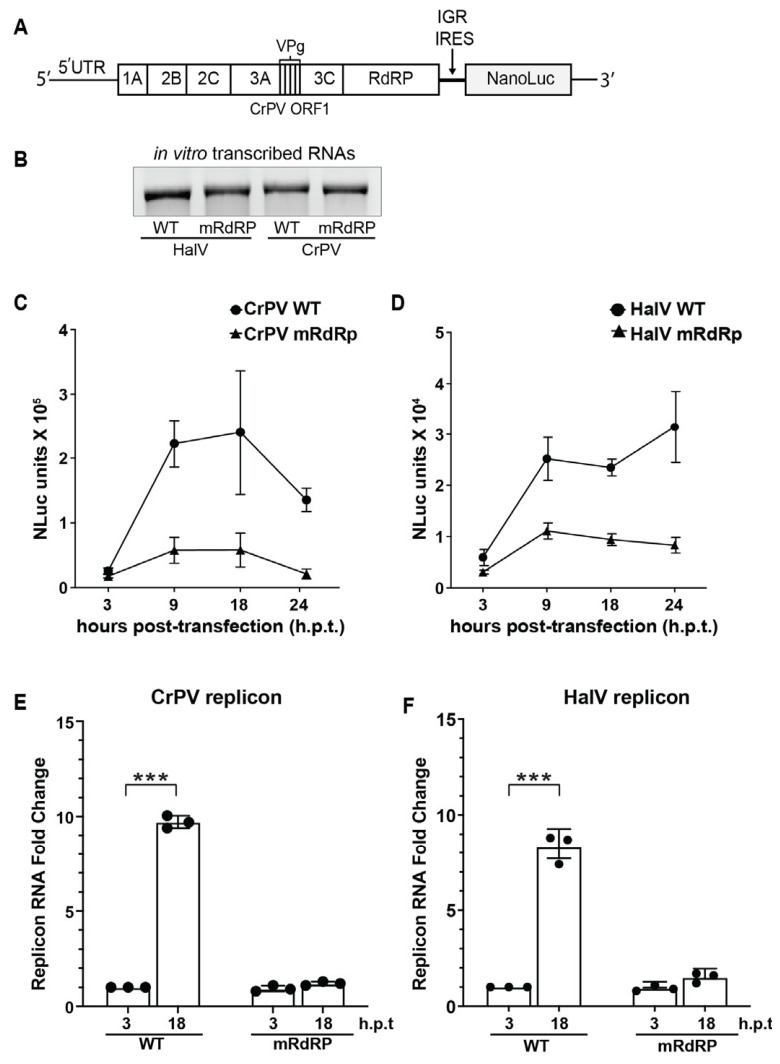
Assessment of HalV IGR IRES activity using a viral replicon system. (**A**) Schematic representation of the CrPV-based replicon system. The structural open reading frame (ORF2) of the CrPV infectious clone was replaced with the Nanoluciferase (NLuc) ORF, driven by the indicated IGR IRES (CrPV or HalV). The AUG start codon of the NLuc gene was removed to ensure translation is strictly IRES-dependent. (**B**) Denaturing agarose gel of in vitro-transcribed replicon wild-type (WT) and RdRp mutant (mRdRp; DD1621-2NN) RNAs. (**C**,**E**) Relative NLuc activities and (**D**,**F**) RNA in S2 cells transfected with the indicated replicons over time. (**E**,**F**) Relative viral RNA levels of wild-type (WT) and RdRP mutant CrPV and HalV replicons in S2 cells quantified by RT-qPCR analysis using eS6 as reference. RT-qPCR fold change (**E**,**F**) was statistically analyzed by paired two-tailed Student’s *t*-test comparing WT and mRdRp RNA levels at each time point (*** *p* < 0.001). Data represent the mean ± s.d. from at least three independent experiments.

Given that HalV IGR IRES can support translation in CrPV-infected cells (Figure 4C), we next examined whether HalV IRES can support translation in a viral replicon. Currently, there is no HalV infectious clone, so we addressed whether the HalV IRES can support a heterologous viral replicon. We engineered an adapted version of a previously established CrPV infectious clone and replicon by replacing the CrPV ORF2 with the Nanoluciferase (NLuc) reporter ORF, which is driven by either the CrPV or HalV IGR IRES (Figure 5A) [36]. We deleted the AUG start codon of NLuc, which ensures translation of NLuc is IGR IRES-dependent. Finally, we generated a replicase-deficient replicon by mutating the catalytic DD1621-2 to NN of the RNA-dependent RNA replicase (RdRP). We in vitro-transcribed the wild-type and RdRP mutant replicons (Figure 5B, Supplemental Figure S1B) and transfected the replicon RNAs in S2 cells and monitored NLuc activity at 3, 9, 18 and 24 h post-transfection. Transfection of the CrPV IGR IRES replicon RNA resulted in robust NLuc activity at 9 and 18 h post-transfection (Figure 5C). In contrast, the RdRP-inactive CrPV replicon displayed significantly lower NLuc activities, indicating that the robust NLuc activities observed with the wild-type replicon are representative of productive viral replication (Figure 5C). Similarly, transfection of the HalV IGR IRES replicon RNA in S2 cells resulted in higher NLuc activities at 9, 18 and 24 h post-transfection (h.p.t.) compared to cells transfected with the RdRP mutant version (Figure 5D). Of note, the CrPV IGR IRES-containing replicon showed ~10-fold more NLuc compared to the replicon containing the HalV IGR IRES, which likely reflects differences in IGR IRES translation efficiencies (Figure 1D and Figure 4). To investigate if the NLuc reporter expression faithfully reflects viral RNA replication, we quantified replicon RNA levels using RT-qPCR. At 18 h.p.t., WT CrPV and HalV RNA levels increased approximately ~9.5-fold and ~8.2-fold over the 3 h baseline, respectively. In contrast, the RdRP mutants exhibited a negligible increase in RNA accumulation at 18 h.p.t. for both replicons, confirming that the observed RNA accumulation is dependent on active viral replication. These results demonstrate that HalV IGR IRES can support the replication of a dicistrovirus replicon.

## 4. Discussion

The HalV IGR IRES (Type 6c) is the most streamlined eukaryotic translation mechanism to date, lacking the major ribosome-binding RNA structural domains (SLIV/SLIV) found in canonical Type 6a and 6b IGR IRESs [46]. In this study, we showed that the HalV IGR IRES can drive translation in insect Sf21 lysates and *Drosophila* S2 cells. We also demonstrated that the HalV IRES can partially support translation in ER-stressed cells and fully direct translation in CrPV infection but not in heat-shocked and serum-starved cells. Finally, we showed that the HalV IGR IRES can support viral replication in a heterologous viral replicon. These findings highlight specific cellular contexts that can support HalV IGR IRES translation.

One of the major puzzles regarding the HalV IGR IRES was the finding of its inactivity in RRLs, previously attributed to the occlusion of the ribosome intersubunit space by the SERBP1/eEF2 complex [46]. Previous cryo-EM structures show that factors like SERBP1 (mammals) and Stm1 (yeast) occupy the mRNA channel and intersubunit space to prevent degradation [53,54,59,60]. Our observation that HalV IRES is active in Sf21 insect lysates and *Drosophila* S2 cells, but not in RRL or wheat germ extract (Figure 1) may suggest that differences in the ribosome composition exist between systems. Orthologous hibernation factors in insects like Lso2 [60], SERBP1 and IFRD2 [54,59] may have lower affinity for the 80S ribosome or may bind in a conformation that does not sterically hinder ribosome assembly on the HalV IRES. Alternatively, the pool of vacant 80S may be more available in insect cells than in RRL.

Even though the mechanistic details of how pre-formed 80S binds to the HalV IRES are still unclear, this phenomenon is not specific to this IRES; the CrPV IGR IRES not only assembles 80S:IRES complexes by first 40S binding then 60S joining but can also bind to pre-formed 80S albeit to a lower extent [61]. Thus, this pathway of ribosome assembly may be more widespread than initially appreciated. It has been known that cellular stresses such as amino acid starvation, osmotic stress, or glucose starvation can lead to the accumulation of non-translating/hibernating 80S ribosomes [54,62,63]. This increased pool of vacant ribosomes could potentially be exploited by the HalV IGR IRES to initiate translation. In this study, we showed that the HalV IGR IRES translation is supported under specific cellular conditions including CrPV infection and partially in ER-stressed cells (Figure 3). Under these cellular conditions when cap-dependent translation is shut off, the increased pool of vacant ribosomes can be usurped by both the HalV and CrPV IGR IRESs to drive translation. However, there may be an additional loss or inactivity of ribosome-associated factors such as SERBP1 and IFRD2 that further allows HalV IGR IRES to function in specific cellular conditions such as during CrPV infection and ER stress but not in heat-shocked or serum-starved cells. It will be important to investigate the ribosome-associated factors and the status of ribosomes during cellular conditions that allow HalV IGR IRES translation especially during CrPV infection, which may provide insights into this viral translation strategy.

The HalV IGR IRES represents an evolutionary trade-off: by discarding the domains required for 40S subunit recruitment, it gained a compact efficiency but might have become strictly dependent on the availability of pre-formed vacant 80S ribosomes. This points to the state of the ribosome as a focal point of regulation for specific IRES translation, which may provide insights into virus restrictions to specific hosts or phases of infection where vacant ribosomes are abundant. Future work identifying the specific factors bound to empty 80S ribosomes in specific species and cellular conditions will be insightful to understand the restrictive nature of this minimal viral RNA element.

## Data Availability

The original contributions presented in this study are included in the article/Supplementary Material. Further inquiries can be directed to the corresponding author.

## Supplementary Materials

The following supporting information can be downloaded at: https://www.mdpi.com/article/10.3390/v18050492/s1. Supplemental Figure S1A, In vitro transcribed capped bicistronic RNA used in Figure 1D, Supplemental Figure S1B, In vitro transcribed replicon RNA used in Figure 5, Supplemental Data S1.
