# Supplementary material for: Functional Analysis of the Halastavi árva Virus (HalV) Internal Ribosome Entry Site
Source: Viruses. 2026 Apr 23;18(5):492. doi: 10.3390/v18050492 (PMC13211661; doi:10.3390/v18050492)
Supplement: Supplementary file 1 [file viruses-18-00492-s001.zip › Chapagain_Revised Supplemental Figure S1.pdf]

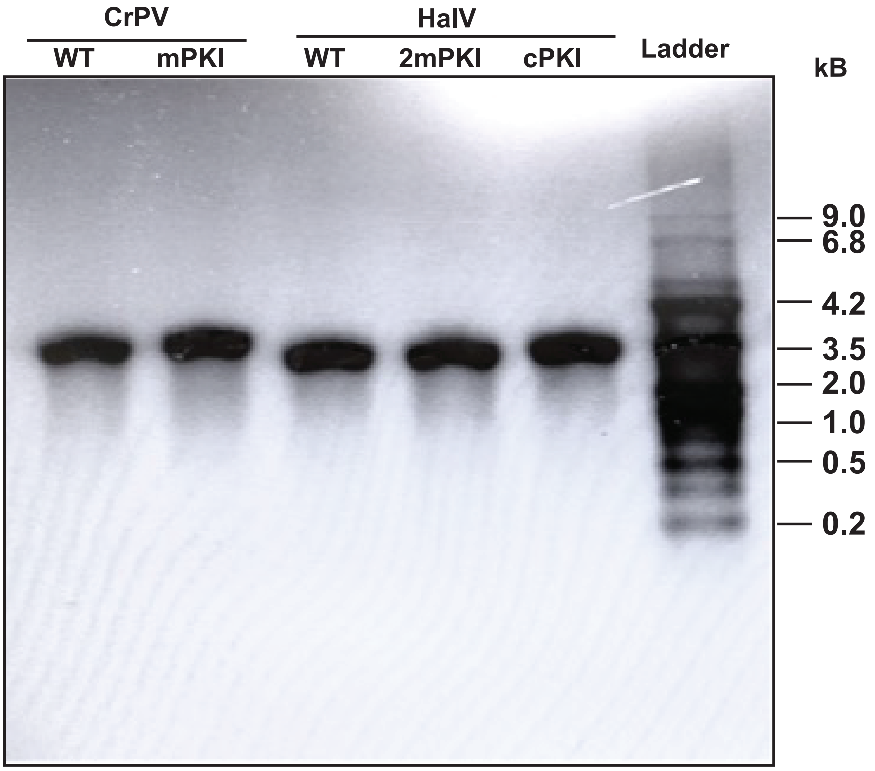

Fig S1A. In vitro transcribed capped bicistronic RNA used in Fig 1D.

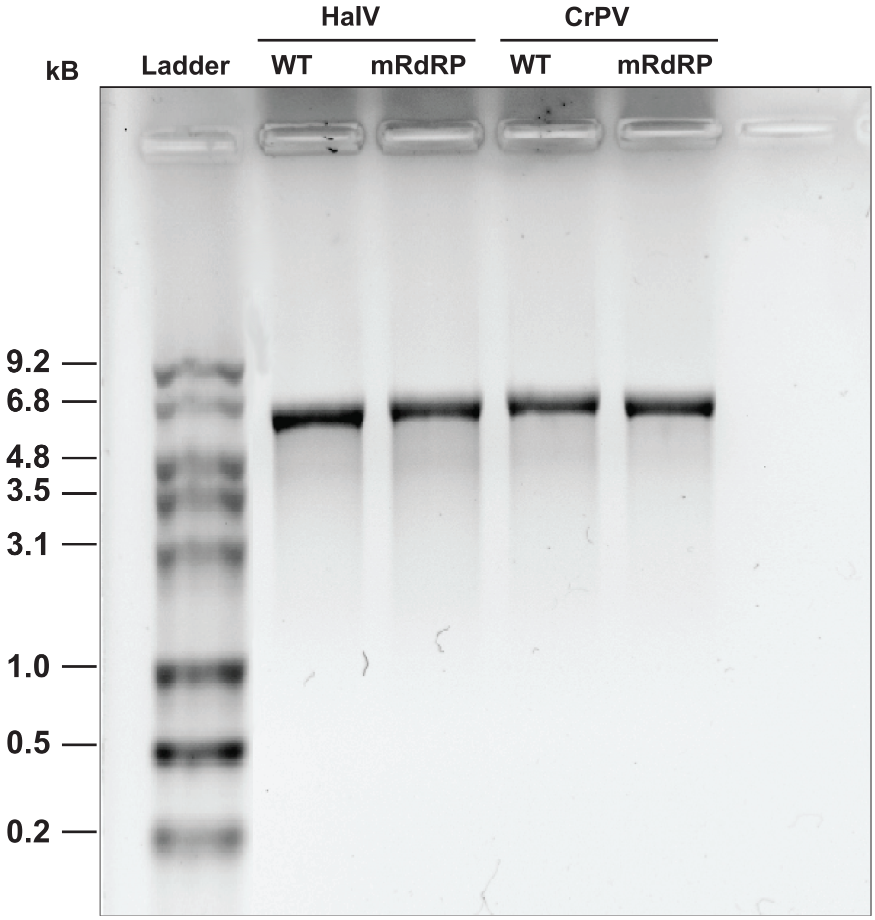

Fig S1B. In vitro transcribed replicon RNA used in Fig. 5.
